# ^187^Os nuclear resonance scattering to explore hyperfine interactions and lattice dynamics for biological applications

**DOI:** 10.1126/sciadv.ads3406

**Published:** 2025-02-07

**Authors:** Iryna Stepanenko, Zhishuo Huang, Liviu Ungur, Dimitrios Bessas, Aleksandr Chumakov, Ilya Sergueev, Gabriel E. Büchel, Abdullah A. Al-Kahtani, Liviu F. Chibotaru, Joshua Telser, Vladimir B. Arion

**Affiliations:** ^1^ University of Vienna, Institute of Inorganic Chemistry, Währinger Strasse 42, 1090 Vienna, Austria.; ^2^ Institute of Science and Technology Austria (ISTA), Am Campus 1, A-3400 Klosterneuburg, Austria.; ^3^ Department of Chemistry, National University of Singapore, Block S8 Level 3, 3 Science Drive 3, Singapore 117543, Singapore.; ^4^ European Synchrotron Radiation Facility, F-38043 Grenoble, France.; ^5^ Deutsches Elektronen Synchrotron, D-22607 Hamburg, Germany.; ^6^ ChemConsult GmbH, P.O. Box 43, 9485 Nendeln, Liechtenstein.; ^7^ Chemistry Department, College of Science, King Saud University, P.O. Box 2455, Riyadh 11451, Saudi Arabia.; ^8^ Theory of Nanomaterials Group, KU Leuven, Celestijnenlaan 200F, B-3001 Leuven, Belgium.; ^9^ Department of Biological, Physical and Health Sciences, Roosevelt University, 430 S. Michigan Avenue, Chicago, IL 60605, USA.

## Abstract

Osmium complexes with osmium in different oxidation states (II, III, IV, and VI) have been reported to exhibit antiproliferative activity in cancer cell lines. Herein, we demonstrate unexplored opportunities offered by ^187^Os nuclear forward scattering (NFS) and nuclear inelastic scattering (NIS) of synchrotron radiation for characterization of hyperfine interactions and lattice dynamics in a benchmark Os(VI) complex, K_2_[OsO_2_(OH)_4_]. We determined the isomer shift [δ = 3.3(1) millimeters per second] relative to [Os^IV^Cl_6_]^2−^ and quadrupole splitting [Δ*E*_Q_ = 12.0(2) millimeters per second] with NFS. We estimated the Lamb-Mössbauer factor [0.80(4)], extracted the density of phonon states, and carried out a thermodynamics characterization using the NIS data combined with first-principles calculations. Overall, we provide evidence that ^187^Os nuclear resonance scattering is a reliable technique for the investigation of hyperfine interactions and Os-specific vibrations in osmium(VI) species and is thus applicable for such measurements in osmium complexes of other oxidation states, including those with anticancer activity such as Os(III) and Os(IV).

## Teaser

^187^Os nuclear resonance scattering allows extraction of Mössbauer parameters in Os complexes such as those of medicinal interest.

## INTRODUCTION

The use of metal complexes as anticancer agents revolutionized cancer treatment more than 50 years ago with the use of cisplatin, *cis*-[PtCl_2_(NH_3_)_2_] (*1*). The efficiency of Pt complexes is, in part, due to their specific kinetics toward ligands substitution and rearrangement, which allows kinetically controlled metal binding to DNA (*2*). Other metal ions of the so-called platinum group (Ru, Rh, Os, Ir, and Au) that have similar ligand-exchange kinetics have also attracted the attention of researchers who hope to increase the efficacy and reduce the side effects and general toxicity of Pt-based drugs. Specifically, ruthenium(III) complexes, namely, KP1019, indazolium *trans*-[tetrachloridobis(1*H*-indazole)ruthenate(III)], BOLD-100 (KP1339, NKP1339, and IT-139), sodium *trans*-[tetrachloridobis(1*H*-indazole)ruthenate(III)], and NAMI-A, imidazolium *trans*-[tetrachlorido(dimethyl sulfoxide)(1*H*-imidazole)] have entered clinical trials for treatment of a broad range of anticancer indications and metastases, respectively (*3*, *4*). This potential for Ru-based drugs has also fueled interest in analogous osmium complexes. The advantage of using osmium analogs with a cytotoxicity similar to that of their ruthenium congeners lies in the higher substitution inertness of osmium species under conditions relevant for drug formulation (*5*, *6*). A family of azole complexes with osmium in different oxidation states (II, III, IV, and VI) (*5*, *7*–*12*) and osmium(VI) complexes with Schiff-base ligands (*13*) have been reported recently, in addition to a large number of organoosmium(II) arene compounds (*14*–*22*), which exhibited varied antiproliferative activity.

The mechanisms of action of many Ru- and Os-based anticancer drug candidates remain unclear. Among other reasons, this is due to a lack of metallodrug speciation data under physiologically relevant conditions (*23*–*25*). Atomic absorption spectroscopy and inductively coupled plasma mass spectrometry are destructive and provide only the total metal amount without information on metal speciation unless individual components are separated (*24*). The complexity of biological fluids makes nondestructive speciation studies using classical spectroscopic methods [nuclear magnetic resonance (NMR), electron paramagnetic resonance (EPR), or ultraviolet-visible spectroscopy] or electrospray ionization mass spectrometry (ESI-MS) challenging. Notable efforts have been made by Walsby and co-workers to apply EPR spectroscopy to the study of clinically relevant Ru(III) complexes under physiological conditions (*26*–*31*). Lay and co-workers demonstrated that x-ray absorption spectroscopy (XAS) is superior for having elemental specificity, being nondestructive to drugs, and having greater tolerance for biologically relevant matrices (*24*). All these advantages make XAS the now preferred technique for the speciation of metallodrugs in biological systems, such as for NAMI-A and KP1019 (*23*, *25*, *32*, *33*).

One disadvantage of XAS (*23*–*25*) [and EPR (*26*, *27*)] is that often higher concentrations than those biologically relevant should be used in the cell medium to obtain a detectable cellular response. Therefore, extrapolation of any such results to biologically relevant concentrations requires additional care as the speciation may change. In this context, the development of element-specific techniques with detection sensitivity at the level of biologically relevant concentrations is desirable.

One element-specific technique, which could be of broad interest is osmium Mössbauer spectroscopy with the same isotopes as for NMR spectroscopy (^187^Os, *I_g_* = 1/2, 1.96% abundance; ^189^Os, *I_g_* = 3/2, 16.156% abundance). However, the short half-life of the radioactive source for ^189^Os Mössbauer spectroscopy, ^189^Ir, is only 13.3 days. This makes such studies for a broader community inconvenient from a practical point of view, and no such sources are now available. The use of ^187^Os isotope for Mössbauer spectroscopy would also be of great interest, but there has never been a suitable radioactive source.

This unfortunate situation was recently lifted by successful excitation (*34*) of the low-lying first excited nuclear level energy state for ^187^Os at 9.778(3) keV (*I_e_* = 3/2) by synchrotron radiation, with demonstration of nuclear forward scattering (NFS) and nuclear inelastic scattering (NIS) feasibility on the ^187^Os metal. This has now paved the way for hyperfine interaction and lattice dynamics characterization of osmium complexes by NFS (*35*, *36*) and NIS (*37*, *38*), namely, experimental extraction of Mӧssbauer parameters and determination of the phonon density of states (DOS). The combination of a low energy of nuclear transition and the large nuclear mass of ^187^Os resulted in a high recoil free fraction, *f*_LM_ = 0.95(1), at room temperature, which makes nuclear resonance scattering highly attractive for investigation of hyperfine interactions and lattice dynamics of osmium complexes.

Herein, we report a benchmark osmium-specific characterization of potassium osmate, K_2_[OsO_2_(OH)_4_], by ^187^Os nuclear resonance scattering of synchrotron radiation. The chemically relevant, characteristic parameters of Mössbauer spectroscopy, namely, the isomer shift (δ) and the nuclear quadrupole splitting (Δ*E*_Q_) are extracted. In addition, the Os-specific vibrational DOS is determined and compared with that obtained by first-principles theoretical calculations, which not only allowed for a precise assignment of vibrational modes containing notable displacements of the Os atom but also verified our first-principles theoretical model. These results show that nuclear resonance scattering of synchrotron radiation applied to ^187^Os species is a powerful tool for measuring local electronic and vibrational properties in osmium complexes, thus offering additional opportunities for speciation studies of osmium-based anticancer drugs.

## RESULTS

**Fig. 1.**
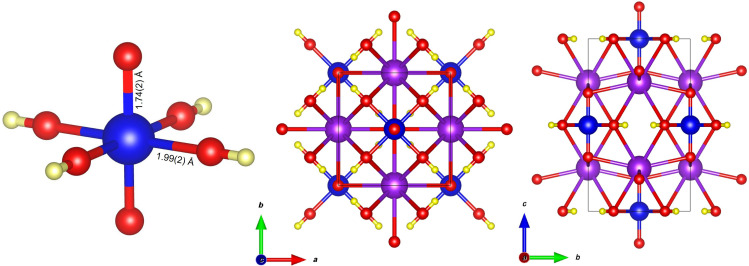
Crystal structure of K_2_[OsO_2_(OH)_4_]. The structure of the anion [OsO_2_(OH)_4_]^2−^ is shown (left) and are projections of the unit cell along axes *c* (middle) and *a* (right). Blue, purple, red, and yellow spheres represent Os, K, O, and H, respectively.

The K_2_[^187^OsO_2_(OH)_4_] sample in this study was prepared as previously reported (*39*), and its identity and purity were confirmed by comparison of unit cell parameters and attenuated total reflectance infrared spectrum (see fig. S1) with the ones reported in the literature (*40*). The crystal structure of K_2_[OsO_2_(OH)_4_] is shown in Fig. 1 (*40*).

The osmium(VI) in the complex is coordinated to four hydroxido ligand oxygen atoms in the equatorial plane at 1.99(2) Å and two oxido ligand oxygen atoms at a shorter interatomic distance [1.75(2) Å] due to strong Os─O π-interactions (*40*).

### Hyperfine interactions

**Fig. 2.**
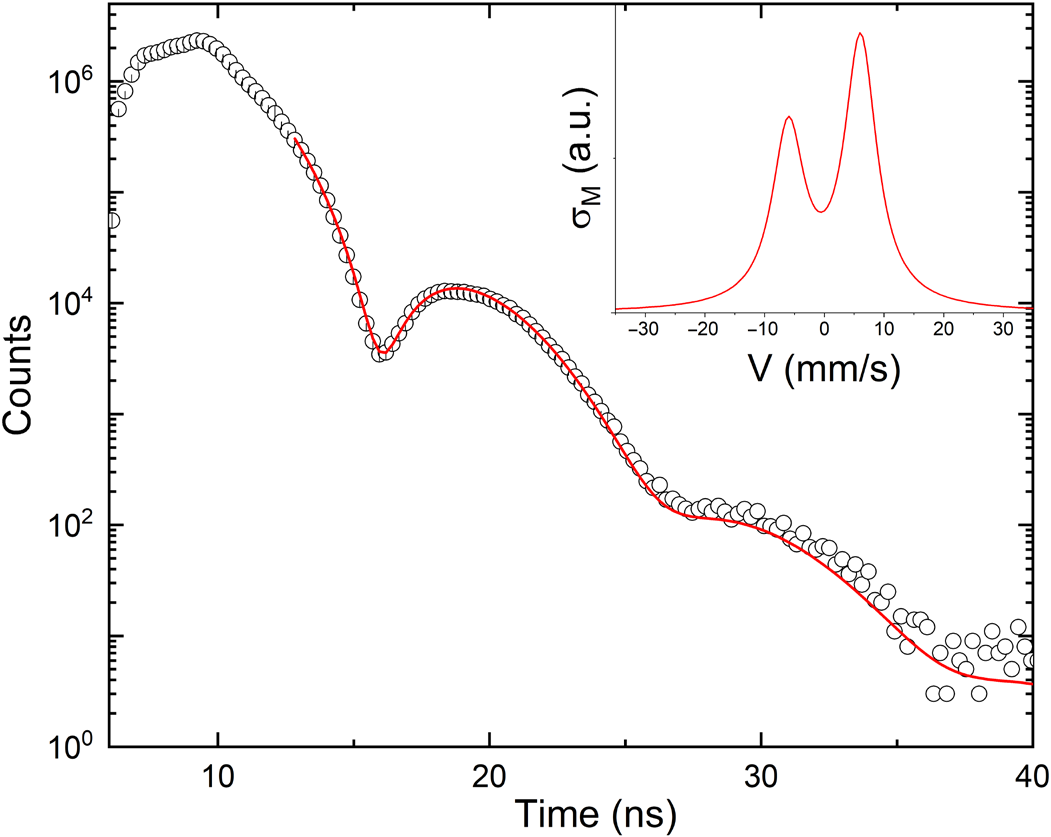
Time evolution of ^187^Os nuclear decay measured with NFS for K_2_[^187^OsO_2_(OH)_4_]. The red curve shows the fit by the CONUSS software. SD, σ, follows Poisson statistics; thus, a typical error bar (not given above) may be calculated as the square root of the recorded intensity. The inset shows simulation of the Mӧssbauer cross section versus Doppler drive velocity corresponding to the fit. a.u., arbitrary units.

We measured the time evolution of the nuclear decay for K_2_[^187^OsO_2_(OH)_4_] by NFS in the time interval between 6 and 41 ns after the prompt pulse of incident synchrotron radiation. A typical count rate for the NFS spectrum was about 1000 photons/s. Such a count rate allows one to measure an NFS spectrum with reasonable statistics in about an hour. The NFS data for K_2_[^187^OsO_2_(OH)_4_] shown in Fig. 2 depict the usual exponential decay superimposed onto clear oscillations with a period of ~10 ns. We fitted the data between 13 and 40 ns using the program CONUSS (*41*, *42*) by a model including a quadrupole hyperfine interaction with a quadrupole splitting, Δ*E*_Q_ = 12.0(2) mm/s, and a texture parameter responsible for the preferential orientation of the electric field gradient direction equal to 39 ± 2%. We did not include the NFS data for times earlier than 13 ns in the fit because they were substantially distorted as the used avalanche photodiode (APD) was heavily overloaded from the prompt x-ray flashes coming from the synchrotron storage ring.

For extracting the isomer shift (δ), an NFS measurement relative to a standard sample is required. In this case, we have chosen (H_2_pz)_2_[^187^Os^IV^Cl_6_] (Hpz = 1*H*-pyrazole) as the standard sample because its Os environment is fully isotropic, i.e., no quadrupole splitting is expected for (H_2_pz)_2_[^187^Os^IV^Cl_6_]. We carried our three NFS measurements in this case: (i) an NFS measurement of K_2_[^187^OsO_2_(OH)_4_] alone, (ii) an NFS measurement of (H_2_pz)_2_[^187^Os^IV^Cl_6_] alone (see fig. S2), and (iii) an NFS measurement of K_2_[^187^OsO_2_(OH)_4_] and (H_2_pz)_2_[^187^Os^IV^Cl_6_] simultaneously (see fig. S3). We paid special attention to measure NFS at the same points of the K_2_[^187^OsO_2_(OH)_4_] and the (H_2_pz)_2_[^187^Os^IV^Cl_6_] samples during the individual and the combined NFS measurements. Because of the absence of an electric field gradient at the position of ^187^Os, the NFS measurement of (H_2_pz)_2_[Os^IV^Cl_6_] shows only a simple exponential decay, without the presence of additional beat(s), which indicates that Δ*E*_Q_ = 0 for (H_2_pz)_2_[Os^IV^Cl_6_], as expected. After extracting all relevant information from the individual NFS measurements, the isomer shift (δ) was the only unknown parameter in the combined measurement. The isomer shift (δ) for K_2_[^187^OsO_2_(OH)_4_] is found to be 3.3(1) mm/s relative to (H_2_pz)_2_[^187^Os^IV^Cl_6_].

### Lattice dynamics

**Fig. 3.**
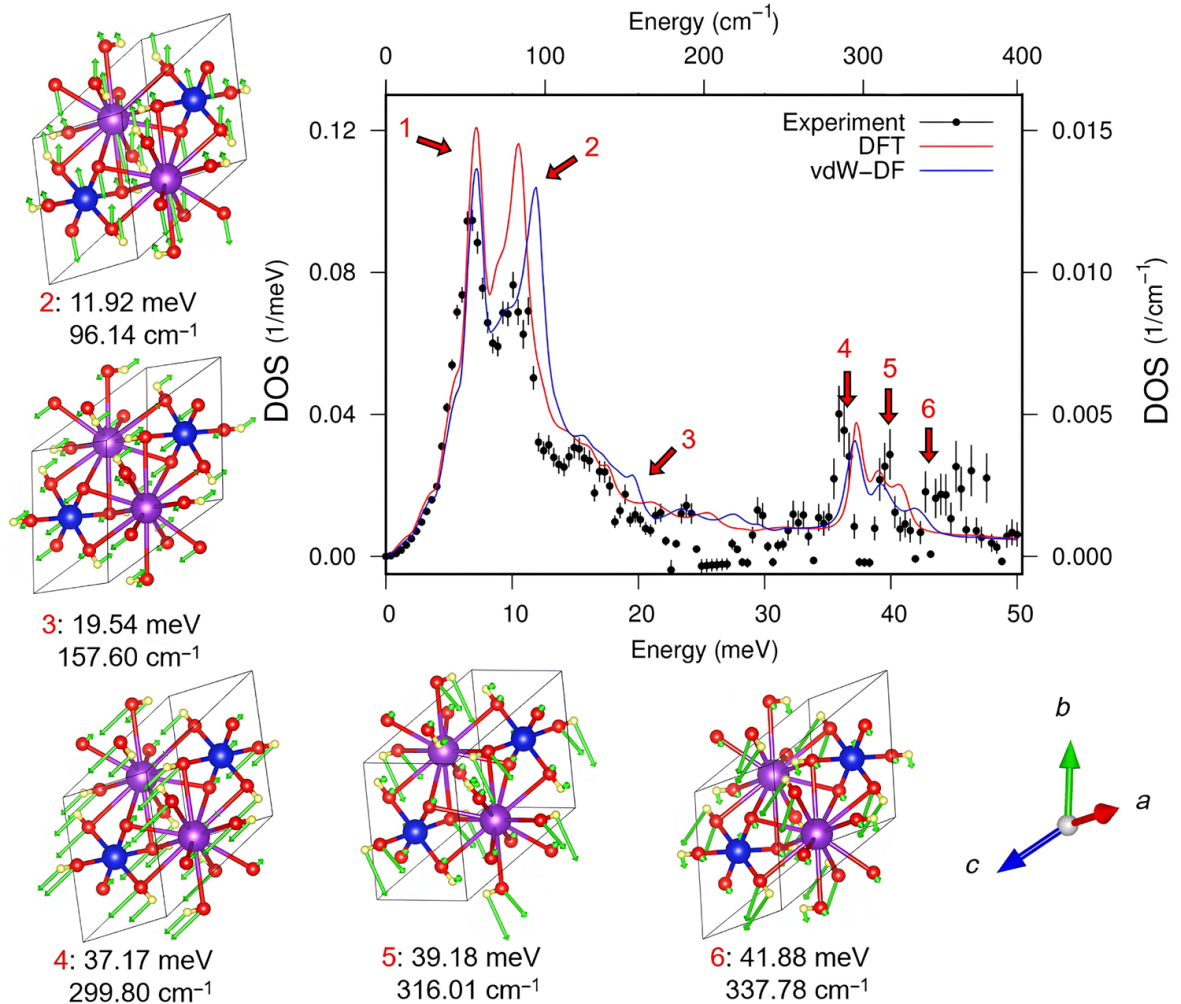
Os-projected phonon DOS of K_2_[^187^OsO_2_(OH)_4_]. The data from NIS are shown as black circles, and the first-principles calculations are shown as lines. Red and blue lines represent DFT calculations with a PBE functional and with additional vdW corrections, respectively. The structural diagrams surrounding the central figure show atomic displacements (green arrows) at each corresponding Os-projected DOS peak (red numbers in DOS plot and displacement diagrams) at the Γ point. Peak 1 corresponds to a single TA mode and thus has no corresponding atomic displacement diagram. The atom coloring scheme is as in Fig. 1: Blue, purple, red, and yellow spheres represent Os, K, O, and H, respectively. The numbers under each plot give the position (energy) of the peak, corresponding to the numbered red arrows in the Os-projected phonon DOS.

We recorded the NIS measurements of K_2_[^187^OsO_2_(OH)_4_] between −10 and +60 meV with respect to the ^187^Os transition energy [9.778(3) keV]. The Os-specific density of vibrational states in K_2_[^187^OsO_2_(OH)_4_] is extracted by using the double Fourier transformation as implemented in the software DOS (*43*). The resulting normalized (to unit area) Os-specific density of vibrational states in K_2_[^187^OsO_2_(OH)_4_] is depicted in Fig. 3.

There are six peaks (for details, see the Supplementary Materials), with two strong ones in the lower-energy region (<12 meV) and four weaker peaks in the higher-energy range (19 to 42 meV). The first peak around 7 meV corresponds to a single transverse acoustic (TA) mode, whereas the other five represent Os-related optical modes. In our theoretical calculations, peaks 2, 3, and 6 show slight differences between density functional theory (DFT) and van der Waals density functional (vdW-DF) methods due to nonlocal interactions, especially in Os+(OH) and K+O vibrations. Peaks 4 and 5, involving localized atom displacements, show similar energies for both methods.

The density of vibrational states, *g*(*E*), provides direct access to a series of thermodynamic parameters (*44*). First, the probability of the recoilless absorption, known as Lamb-Mӧssbauer factor, can be extracted using Eq. 1

$$f_{\text{LM}}=\text{exp}\left[-E_{R}\int g\left(E\right)\left(\frac{1+e^{-\mathrm{\beta E}}}{1-e^{-\mathrm{\beta E}}}\right)\frac{dE}{E}\right]$$ (1)

where *E*_R_ = 0.274 meV, is the recoil energy for an ^187^Os isolated nucleus and β = 1/*k*_B_*T*, where *k*_B_ is the Boltzmann constant and *T* is the temperature at which *g*(*E*) is measured. The calculated *f*_LM_ for Os in K_2_[^187^OsO_2_(OH)_4_] is 0.80(4) at room temperature. From the Lamb-Mӧssbauer factor, the purely incoherent mean square atomic displacement parameter, 〈*u*^2^_Os_〉, is given by

$$\langle {u^{2}}_{\text{Os}}\rangle =\frac{-\text{ln}\left(f_{\text{LM}}\right)}{k^{2}}$$ (2)

where *k* = 4.959 Å^−1^ is the wave number of the resonant photons. The Os mean square atomic displacement in K_2_[^187^OsO_2_(OH)_4_] calculated in this way is 〈*u*^2^_Os_〉 = 90(10) pm^2^ at room temperature. Note that the mean square Os displacement obtained from the thermal parameters of the single-crystal x-ray diffraction (scXRD) structures measured by others (*40*) and by us are larger, despite being measured at lower temperatures: 111(7) pm^2^ at 173 K (*40*) and 173(5) pm^2^ at 247 K (this work), respectively. As noted earlier in a study of Zintl phase materials (FeSb_3_ and YbFe_4_Sb_12_) (*45*, *46*), the displacement parameter 〈*u*^2^_M_〉 (M = ^187^Os here and ^57^Fe in the previous works) determined by NIS is much smaller than that determined by scXRD. As stated by Möchel [ref. (*46*), p. 47], the displacement parameter obtained by NIS “is not affected by the site occupation or disorder as the 〈*u*^2^〉 values obtained from diffraction sometimes are affected.”

The Os-specific mean-force constant, 〈*F*_Os_〉, is obtained from Eq. 3

$$\langle F_{\text{Os}}\rangle =M\int \frac{g\left(E\right)E^{2}dE}{\hbar^{2}}$$ (3)

where *M* is the mass of the resonant isotope, 187 u. The obtained value for K_2_[^187^OsO_2_(OH)_4_] is 344(40) N/m. In addition, the vibrational DOS gives the vibrational entropy, *S*^vib^_Os_, using hyperbolic trigonometric functions as given by Eq. 4

$$S_{\text{Os}}^{\text{vib}}=3k_{B}\int_{0}^{\infty }g\left(E\right)\left(\left\{\frac{\mathrm{\beta E}}{2}\text{coth}\left(\frac{\mathrm{\beta E}}{2}\right)-\text{ln}\left[2\text{sinh}\left(\frac{\mathrm{\beta E}}{2}\right)\right]\right\}\right)dE$$ (4)

The contribution of the Os-specific vibrations to the total entropy of the system is 5.0(2) *k*_B_. The calculated Os-projected phonon DOS is shown in Fig. 3 by blue and red lines corresponding to the two used calculation methods. To facilitate comparison with experimental data (black circles in Fig. 3), we convoluted the calculated Os-projected phonon DOS (figs. S4 and S5) with the instrumental function of the used high-resolution monochromator. The corresponding atomic displacements at the Γ point, which is the highest symmetry point (the zero-momentum transfer point; also known as the center of the Brillouin zone), are shown in the lateral plots in Fig. 3.

Figure S4 of the Supplementary Materials shows the expected existence of some contributions to the Os-partial DOS from modes at ~66 meV (~530 cm^−1^) and ~100 meV (~800 cm^−1^) (see also fig. S1). Measurement of these modes is not practical as it would require a >600 times longer acquisition time. Our estimations of the dependence of the calculated parameters from these higher-energy modes show that the calculated parameters of the Lamb-Mössbauer factor, mean square displacements, and entropy are not affected by the higher-energy modes within the quoted error bars. In contrast, the parameter of the force constant strongly depends on the contribution of the higher-energy modes. Therefore, the quoted force constant value calculated by Eq. 3 is valid only for the vibrational modes with energies below 50 meV (~400 cm^−1^).

## DISCUSSION

### Analytical methodologies for Ru- and Os-containing systems with detection sensitivity relevant to biological applications

XAS is now one of the most successful techniques for investigation of transformation of prodrugs in biological fluids (*23*–*25*, *32*, *33*). High sensitivities at micromolar concentrations have been achieved in modern XAS studies (*47*, *48*). In addition to XAS, synchrotron-based x-ray fluorescence (XRF) spectroscopy has been found recently to be a valuable tool for the investigation of biotransformations of anticancer ruthenium(III) and organoosmium(II) complexes (*25*, *49*). NMR techniques, such as Ru or Os NMR and Mӧssbauer spectroscopies, have been used even more rarely, if at all. Multinuclear NMR is a powerful technique for investigating metal-based drug speciation under physiologically relevant conditions in the case of diamagnetic species (e.g., low-spin d^6^ complexes) (*50*). Direct observation of NMR resonances for the metals themselves is potentially very useful but often difficult to achieve. Neither of the two magnetically active isotopes of Os, ^187^Os (*I* = 1/2) and ^189^Os (*I* = 3/2) with 1.96 and 16.15% natural abundances, respectively (see https://webelements.com/osmium/isotopes.html), provide a reasonable NMR signal. Indirect detection of ^187^Os nuclear resonances by polarization transfer techniques from sensitive nuclei, ^1^H and ^31^P, has been previously reported (*51*) and provides a great increase in sensitivity of ^187^Os detection. Sharp ^1^H and ^31^P NMR resonances were reported for a number of organoosmium and other complexes (*52*–*55*). Isotopic enrichment to more than 98% allowed straightforward detection of the ^187^Os resonance in an osmium-arene complex (*50*).

In contrast to NMR or EPR, Mössbauer spectroscopy can be applied to species with any number of d electrons, even, odd, or none. This technique is highly effective in determining the electronic structure and oxidation state of iron complexes, using ^57^Fe (*56*). Further within group 8, examples of ^99^Ru (*I* = 5/2, 12.76% abundance) Mössbauer investigations are well documented (*56*–*60*), but radiochemical (cyclotron) facilities for the ^99^Rh precursor (half-life of 16 days) are now unavailable and the high energy (89.36 keV) is a complicating issue in the use of synchrotron radiation (*61*). Thus, ^99^Ru Mössbauer is not currently available to investigate the many ruthenium complexes of direct anticancer relevance. ^189^Os Mössbauer spectra were successfully measured in the past for a series of 15 osmium compounds in oxidation states +8, +6, +4, +3, and +2 by using the best-suited 36.2- and 69.5-keV nuclear transitions (*62*). However, at that early date, no Os(II), Os(III), Os(IV), and Os(VI) coordination complexes of relevance as anticancer agents were studied; this work was done before the introduction even of Pt-based anticancer drugs. Nowadays, radioactive sources relevant to the excitation of the 36.2- and 69.5-keV nuclear transitions are not widely available but synchrotron radiation for excitation of the 9.778(3)-keV nuclear level of ^187^Os may be used.

In summary, from the current widely available analytical methodologies relevant to Os-containing systems, it is only XAS/XRF and nuclear resonance scattering that may provide direct access in determining the electronic structure and the oxidation state of osmium complexes.

### Hyperfine interaction characterization using NFS

It is well documented that the isomer shifts (δ) for Fe, Ru, Os, Ir, Pt, and Au compounds are dependent on the oxidation state of the transition metal. The isomer shift is the product of a nuclear term, namely, the change in nuclear radius between the excited and ground states, Δ〈*r*^2^〉, and an electronic term that is the difference in s-electron density at the nucleus between the sample and a reference material. Generally, an increase in s-electron density at the nucleus is related to an increase in oxidation state of the transition metal, which diminishes the deshielding effect of the remaining valence d electrons. Therefore, for ^57^Fe, ^189^Os, and ^195^Pt, where Δ〈*r*^2^〉 is negative, the isomer shift normally decreases with oxidation state, whereas for ^99^Ru, ^193^Ir, and ^197^Au with positive Δ〈*r*^2^〉, δ usually increases (*63*, *64*). Here, we found δ for K_2_[^187^Os^VI^O_2_(OH)_4_] to be +3.3(1) mm/s versus H_2_pz[^187^Os^IV^Cl_6_], which was used as a convenient standard for our systems of interest. Thus, according to the general trend, our results would indicate that Δ〈*r*^2^〉 is positive for ^187^Os. However, the general trend can be affected if ligands with back-bonding abilities, such as CO, CN^−^, or NO^+^, are involved in coordination to the metal. Transfer of electron density from d orbitals to empty π* orbitals of the ligand can result in an increase in s-electron density on the nucleus, similar to what is seen with a higher formal oxidation state. The δ is also useful in determination of electron shielding and the electron-withdrawing power of electronegative groups on the ligands. We plan to expand the range of Os oxidation states in future work, allowing correlation of ^187^Os isomer shift with coordination chemistry.

The second parameter extracted from our analysis is the quadrupole splitting (Δ*E*_Q_), which can be useful for identification of spin state, site symmetry, and the arrangement of ligands. The quadrupole splitting arises from the interaction of a nonspherical nuclear charge distribution (*I* > 1/2) with the electronic electric field gradient, whose sign, strength, and asymmetry depend on the ligand environment of the nuclei. The quadrupole splitting of K_2_[^187^OsO_2_(OH)_4_], Δ*E*_Q_ = 12.0(2) mm/s, clearly indicates that the Os local environment is anisotropic, as expected for this strongly axial system, wherein the axial electric field from the two oxido ligands differs greatly from the equatorial field from the four hydroxido ligands. These two unique Mӧssbauer parameters can now be easily obtained for ^187^Os using NFS and used for identification of particular species by comparison to model compounds.

### Lattice dynamics characterization using NIS

Herein, we apply nuclear resonance scattering not only for investigating the hyperfine interactions between the electronic cloud and the ^187^Os nucleus, encoded in the isomer shift and quadrupole splitting in K_2_[^187^OsO_2_(OH)_4_], but also to determine the Os-specific vibrations.

Because Os is the heaviest atom in K_2_[^187^OsO_2_(OH)_4_], in accordance with the mass homology relation (*65*), it mainly contributes to the lower-energy phonon branches, i.e., energies not exceeding 50 meV (see fig. S5), although the entire phonon spectrum extends up to energies of >400 meV (see fig. S4).

One should note here that, besides Γ, the highest symmetry point, other symmetry points of the Brillouin zone contribute to the peaks due to the extreme nature of phonon dispersion in these lower symmetry points. Compared to calculations of vibrations in isolated molecules (*66*), phonon calculations give a more realistic picture of atomic displacements at the Γ point because they take into account the interactions among molecular units in the crystal, which is especially important for low-frequency optical modes (*67*). Notably, the calculated phonon DOS at 0 K matches well the experimental one measured at room temperature. The same effect is also quantified in a recoil free fraction, *f*_LM_, of about 0.8 at room temperature extracted from our NIS measurements. Comparison of the two theoretical approaches we used in this study, DFT and vdW-DF, shows that the latter method gives better agreement with experiment. Not only did we carry out an osmium-specific thermodynamics characterization for K_2_[^187^OsO_2_(OH)_4_] (the purely incoherent mean square osmium displacement parameter, the Os-specific mean-force constant, and the Os vibrational entropy were extracted), but also we developed a model for this complex based on first principles and verified its vibrational part using NIS. Such a model can readily be used for predicting the microscopic interactions of Os in osmium-based anticancer drugs, thus offering additional opportunities for speciation studies.

In summary, we investigated NFS and NIS of synchrotron radiation by the low-lying nuclear level of ^187^Os [9.778(3) keV] in K_2_[^187^OsO_2_(OH)_4_]. We determined the isomer shift, δ, and quadrupole splitting, Δ*E*_Q_, by fitting the experimental NFS spectra. These results show that ^187^Os NFS is a viable technique for investigation of hyperfine interactions in an osmium(VI) compound, so this approach can be extended to complexes of Os(VIII), Os(IV), Os(III), and Os(II) as well as to other Os(VI) complexes of interest such as osmium(VI)-nitrido complexes with four equatorial chlorido ligands, which are medicinally more relevant than the hydroxido ligands in the current complex. NIS performed with a 1-meV resolution allowed extraction of the density of phonon states, Os-pDOS, which, in combination with first-principles phonon calculations, allowed us to fully model the K_2_[^187^OsO_2_(OH)_4_] system both from the electronic and the atomic vibration perspective.

We expect that ^187^Os NFS, whether combined with XAS, will open an avenue for investigation of biotransformations of osmium-based prodrugs in “real-world” environments, namely, cell culture media, the extracellular matrix, the cellular cytoplasm, and the cell nucleus. This approach will provide deeper insights into the underlying mechanisms of the anticancer activities of Os complexes due to the sensitivity of Mössbauer parameters to changes in oxidation state and coordination environment with an instrumentation sensitivity comparable to modern XAS techniques. As in the case of XAS speciation studies on Ru prodrugs (*25*, *32*), ^187^Os NFS data obtained for a library of model five- and six-coordinate Os complexes in different oxidation states and with different donor atom coordination environment (for benchmarking of the δ and Δ*E*_Q_ parameters) are required before speciation studies for potential osmium-based anticancer drugs could be performed.

## MATERIALS AND METHODS

### Synthesis

We prepared K_2_[^187^OsO_2_(OH)_4_] from ^187^Os metal (99.55% enriched, obtained from SC “PA Electrochemical Plant”, Russia), which was converted to ^187^OsO_4_ by heating in a quartz tube in a stream of air. We reacted the enriched osmium tetroxide with 6 M aqueous KOH followed by addition of ethanol as a reductant (*39*). Crystallization in air afforded XRD-quality single crystals. We determined cell parameters at 247 K for a tetragonal crystal, *I*4/*mmm* [*a* = *b* = 5.5904(2), *c* = 9.4276(4) Å], which were in line with those previously reported (*40*). The three strong IR absorption bands at 3281, 1105, and 795 cm^−1^ (fig. S1) are in good agreement with those reported for the same complex with naturally abundant osmium (*40*), providing further evidence for sample purity. The negative-ion ESI mass spectrum provides further evidence of its identity. A peak with mass/charge ratio (*m*/*z*) 328.88 could be assigned to the ion pair {K^+^[^187^OsO_2_(OH)_4_]^2−^}^−^ (fig. S6). We prepared (H_2_pz)_2_[^187^OsCl_6_] as an orange powder by reaction of [(dimethyl sulfoxide)_2_H]_2_[^187^OsCl_6_], which was obtained directly from ^187^OsO_4_ (*9*), in dry ethanol with an excess of 1*H*-pyrazole at room temperature as reported previously (*11*). The negative-ion ESI mass spectrum showed a peak with *m*/*z* 363.75, which could be attributed to [^187^Os^IV^Cl_5_]^−^ (see fig. S7).

### Sample preparation

We spread the polycrystalline K_2_[^187^OsO_2_(OH)_4_] (~8 mg) over an area of 10 mm^2^ and enclosed it in Kapton tape.

### X-ray crystallography

We performed the measurement on a Bruker D8 Venture diffractometer and processed the data using the SAINT software (*68*). Crystal data, data collection parameters, and structure refinement details are given in table S1. We solved the structure by direct methods and refined by full-matrix least-squares techniques. Non-H atoms were refined with anisotropic displacement parameters. The H atom position was found from difference Fourier map, and its positional parameters were refined according to the geometry of the H-bond. The following computer programs and hardware were used: structure solution, SHELXS-2014 and refinement, SHELXL-2014 (*69*); molecular diagrams, ORTEP (*70*); and computer, Intel CoreDuo.

### Hyperfine interaction and lattice dynamics characterization

We carried out nuclear resonance scattering measurements, both NFS and NIS, at nuclear resonance beamlines ID18 (*71*) and ID14 of the European Synchrotron Radiation Facility (ESRF), Grenoble, France. The synchrotron radiation storage ring was operating in 16-bunch mode providing x-ray flashes every 176 ns. The optical elements used at the nuclear resonance energy of ^187^Os, 9.778(3) keV, have been described recently (*34*). The monochromatic flux incident to the sample was 2.8 × 10^9^ photons s^−1^ meV^−1^ at a 90-mA current in the synchrotron storage ring. We used sets of APDs for acquiring both the NFS and NIS spectra. For NFS, we have used a 5 mm–by–5 mm–by–100 μm APD and, for NIS, a 10 mm–by–10 mm–by–100 μm APD. The estimated solid angle captured in the NIS case was about 0.8 × 2π sr (the sample was about 1 mm away from the APD). A typical count rate on the elastic line of the inelastic spectrum was about 100 counts/s; at the first peak of the inelastic spectrum at around 10 meV, it was about 5 counts/s. The total acquisition time for NIS measurement shown in this study was about 9 hours, and for the NFS measurements, it was about 3000 s. Both NFS and NIS measurements were carried out under ambient conditions. We placed the samples (see above) on motorized stages, and a region with a thickness that corresponds to approximately one electronic absorption length was identified and chosen for the NFS measurements.

### Computational methods

We performed all electronic and phonon structure calculations using the Quantum ESPRESSO software (*72*, *73*) with the revised Perdew-Burke-Ernzerhof (PBE) functional (*74*) and optimized norm-conserving Vanderbilt pseudopotentials (*75*) taken from the pseudopotentials PSEUDO DOJO (*76*). We set the plane-wave kinetic energy cutoff and the density cutoff to 1360 and 5442 eV, respectively, and used a shifted 8 × 8 × 8 Monkhorst-Pack mesh for Brillouin zone integration to ensure convergence. The convergence threshold of total energy was 1.3 × 10^−11^ eV for self-consistent field calculations. The experimental structure was fully relaxed with the force on each atom smaller than convergence and the total energy convergence threshold set to 2.6 × 10^−4^ eV/Å and 1.3 × 10^−13^ eV, respectively. We performed phonon calculations (figs. S4 and S5) with density functional perturbation theory (*77*), in which a 2 × 2 × 2 mesh for Brillouin zone integration and 1.3 × 10^−15^ eV for the self-consistent threshold were used. The projected phonon DOS calculation was carried out with a 28 × 28 × 28 mesh to guarantee convergence. Because of the existence of Os─OH bonding in the ion [OsO_2_(OH)_4_]^2−^, the material can be considered as a pseudo-molecular crystal; thus, the nonlocal interaction (vdW) should be taken into consideration, which is performed by the vdW-DF method (*78*, *79*).
