# Supplementary material for: 187Os nuclear resonance scattering to explore hyperfine interactions and lattice dynamics for biological applications
Source: Sci Adv. 2025 Feb 7;11(6):eads3406. doi: 10.1126/sciadv.ads3406 (PMC11804912; doi:10.1126/sciadv.ads3406)
Supplement: Supplementary file 1 — Supplementary Text Figs. S1 to S7 Table S1 [file sciadv.ads3406_sm.pdf]

Science Advances
AAAS

Supplementary Materials for

^{187}Os nuclear resonance scattering to explore hyperfine interactions and lattice dynamics for biological applications

Iryna Stepanenko *et al.*

Corresponding author: Vladimir B. Arion, vladimir.arion@univie.ac.at;
Liviu F. Chibotaru, Liviu.Chibotaru@kuleuven.be; Joshua Telser, jtelser@roosevelt.edu

Sci. Adv. **11**, eads3406 (2025)
DOI: 10.1126/sciadv.ads3406

This PDF file includes:

Supplementary Text
Figs. S1 to S7
Table S1

1. Detail to Figure 3 in the main text

We can see that there are in total six peaks, of which two peaks are in the lower-energy region (<12 meV) with much higher strength than the four peaks in the higher energy region (19–42 meV). This is because of the large atomic mass difference between Os and the other atoms. The first peak shown in Figure 3 corresponds to a single transverse acoustic (TA) mode. The other five peaks represent Os-involved optical modes with the representative vibrations shown as the surrounding subplot in Figure 3. Peak 2 corresponds to the vibration between the group of Os+(OH) and that of K+O and also shows contributions from longitudinal acoustic (LA) and second TA modes. Peak 3 corresponds to the displacement of the group of Os+O in (OH) relative to the group K+O+H in (OH). Peak 4 involves the vibration between the group of Os+K+O and OH. Peaks 5 and 6 correspond to vibrations involving the three groups: Os+K, O+(OH), and (OH). Peaks 2, 3, and 6 calculated by DFT differ slightly from those calculated by the vdW+DF method. Such a difference is reasonable, as vdW+DF considers non-local interaction corrections. Given the presence of OH groups in $K_2[^{187}OsO_2(OH)_4]$, the effect of van-der-Waals interactions was taken into account and delivered a slightly better agreement with experiment. Peak 1 does not involve sensible relative displacements of nearby atoms, thus the two methods predict the same peak energy. For Peak 2, since the two groups involved in vibration, Os+(OH) and K+O, have a largely spaced distribution of charge, the non-local interaction makes a shift of about 2 meV. The situation with Peaks 3 and 6 is as with Peak 2, involving additional local vibration of O-H. Peak 4 corresponds to the displacements of individual atoms (Os, K, and O) and (OH) groups, all having localized charge distributions, therefore, DFT and vdW-DF calculations give a similar result, which is alike to the situation of Peak 5.

2. IR spectra

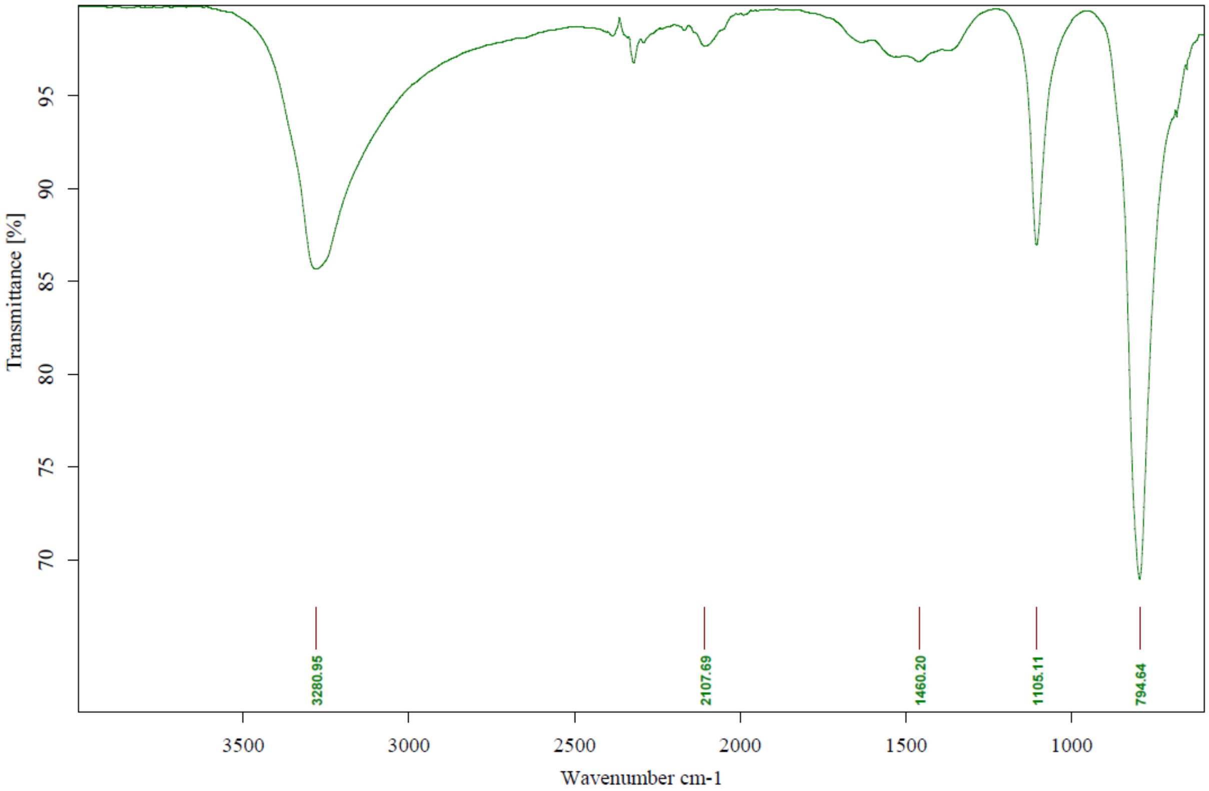

Figure S1. The IR spectrum of $K_2[^{187}Os^{VI}O_2(OH)_4]$.
The strongest bands (3281 cm^{-1}, 1105 cm^{-1}, and 794.6 cm^{-1}) correspond closely to those reported by Murmann and Barnes: 3300 cm^{-1}, 1070 cm^{-1}, and 803 cm^{-1} *(40)*. The first of these is an asymmetric OH stretch (E_u symmetry in idealized D_{4h} point group symmetry) and the latter two are likely asymmetric Os-O(ax) (A_{2u}) and Os-O(eq) (E_u) stretches.

3. NFS spectra

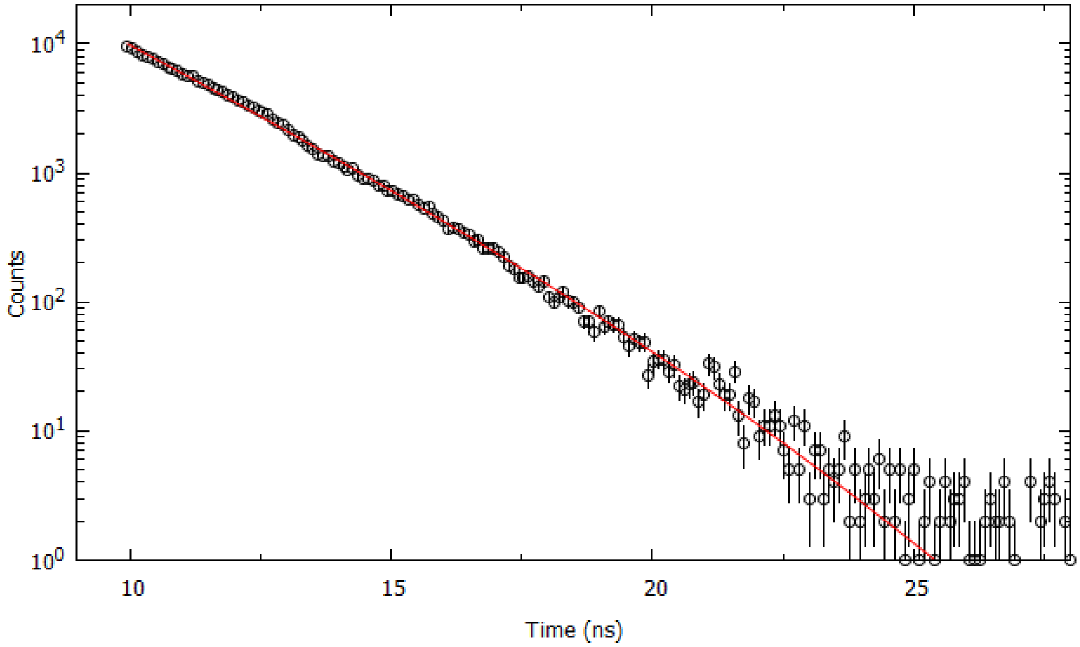

Figure S2. NFS spectrum of $(H_2pz)_2[^{187}OsCl_6]$.

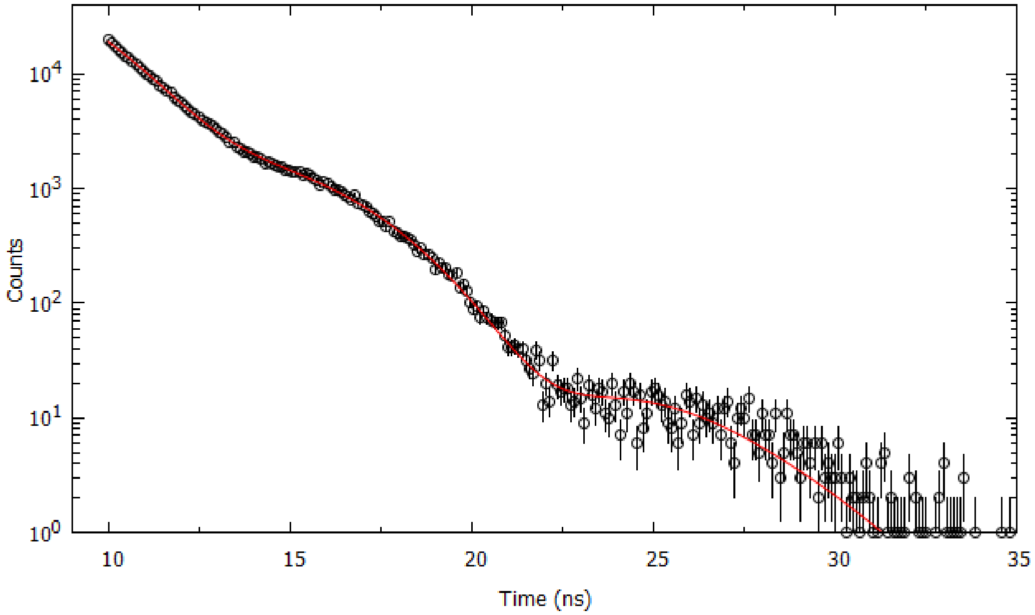

Figure S3. Combined NFS spectrum of $(H_2pz)_2[^{187}OsCl_6]$ and $K_2[^{187}Os^{VI}O_2(OH)_4]$.

4. Phonon dispersion (left) and phonon DOS (right) in $K_2[^{187}OsO_2(OH)_4]$

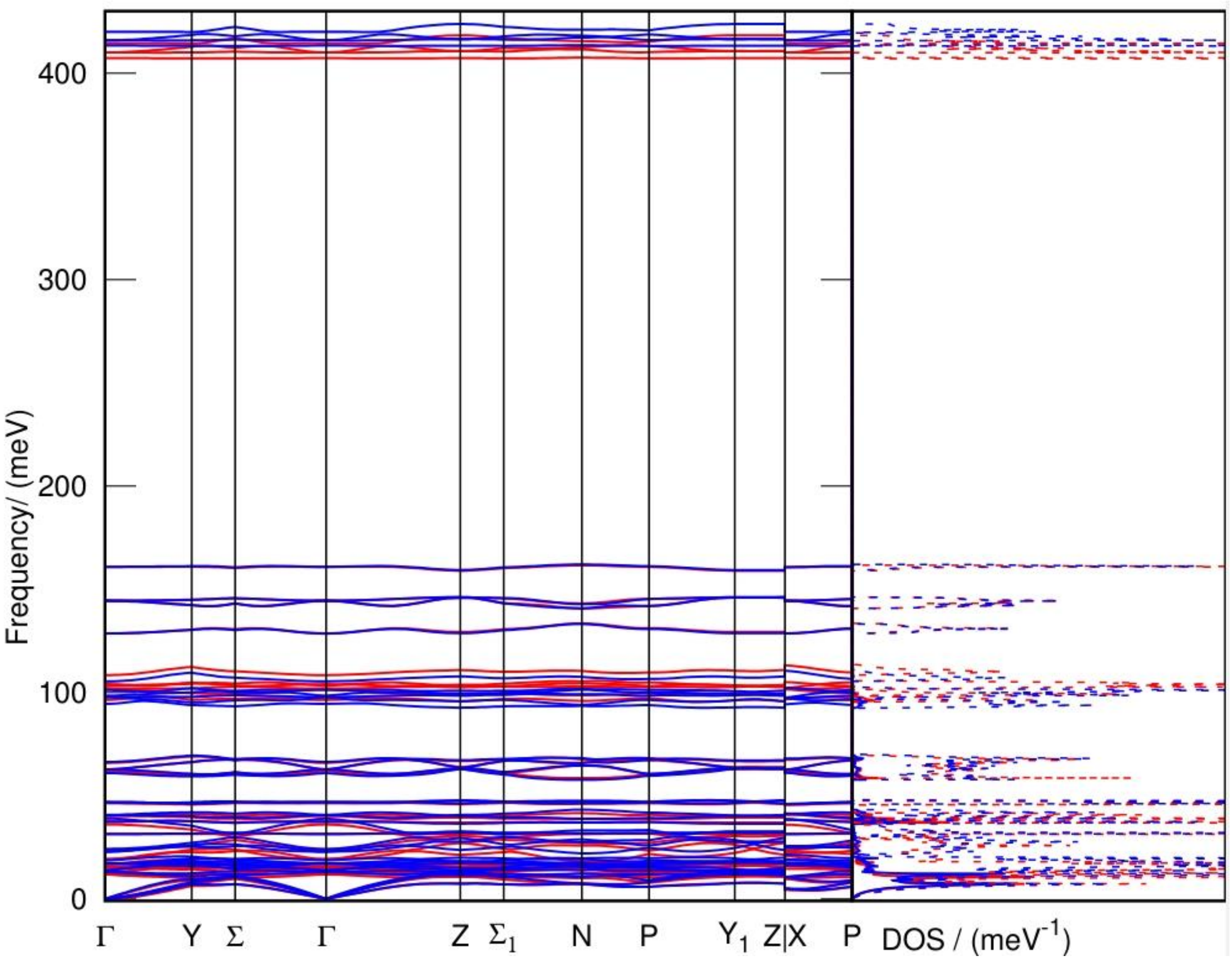

Figure S4. The phonon dispersion and phonon DOS in $K_2[^{187}OsO_2(OH)_4]$.
Red and blue lines correspond to DFT calculations with PBE functional and with additional vdW-DF corrections, respectively (see Computational Methods in the main text). Solid and dashed lines in the right plot represent the Os-projected DOS and total DOS, respectively.

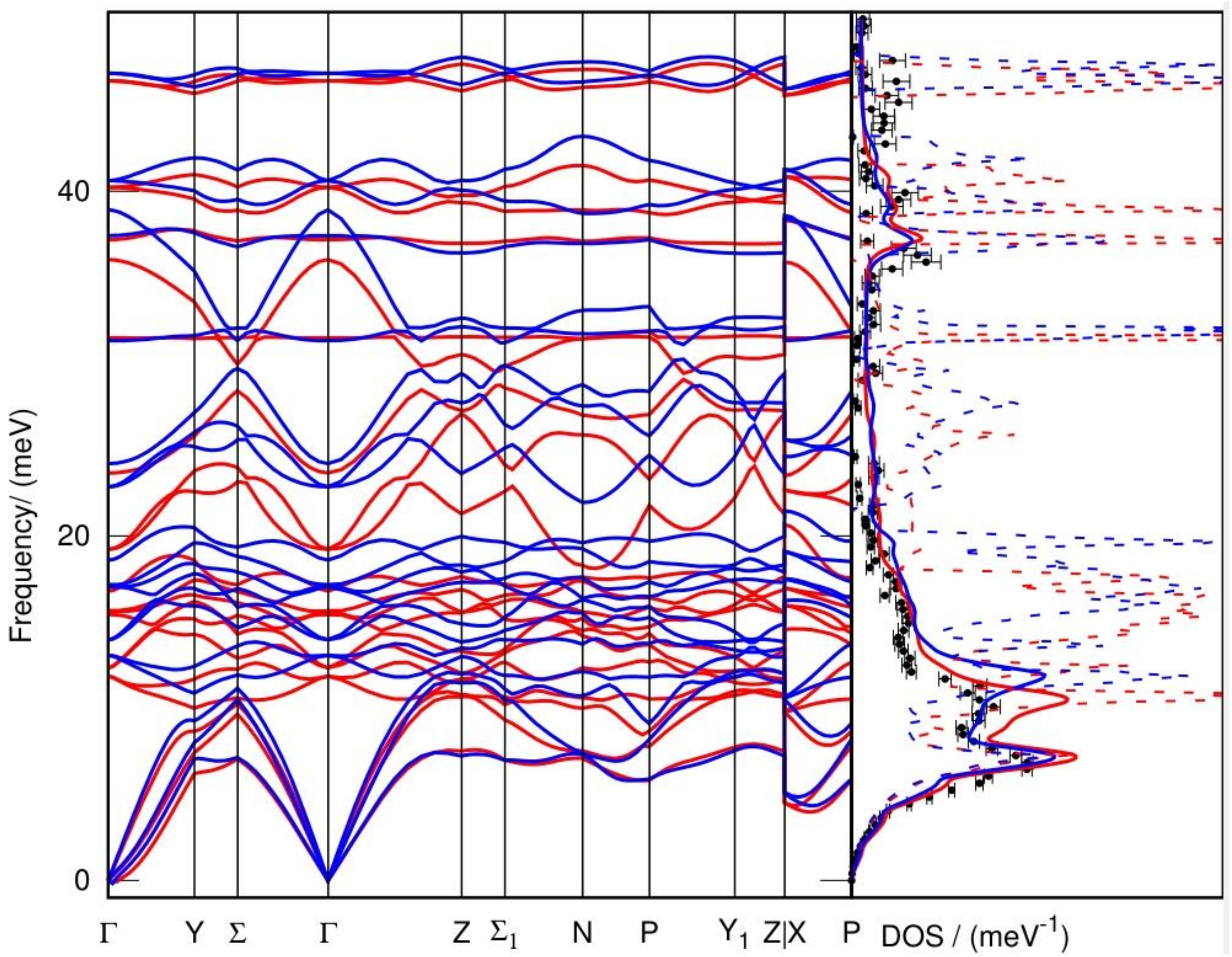

Figure S5. The phonon dispersion in low-energy domain and the corresponding Os-projected phonon DOS convoluted with the instrumental resolution density of states.

Red and blue lines have the same meaning as in Figure S4. The full phonon dispersion and the convoluted Os-projected DOS with the instrumental resolution density of states are shown with solid lines. For comparison, the total DOS is shown in the right plot with dashed lines.

5. Negative ion ESI mass spectra

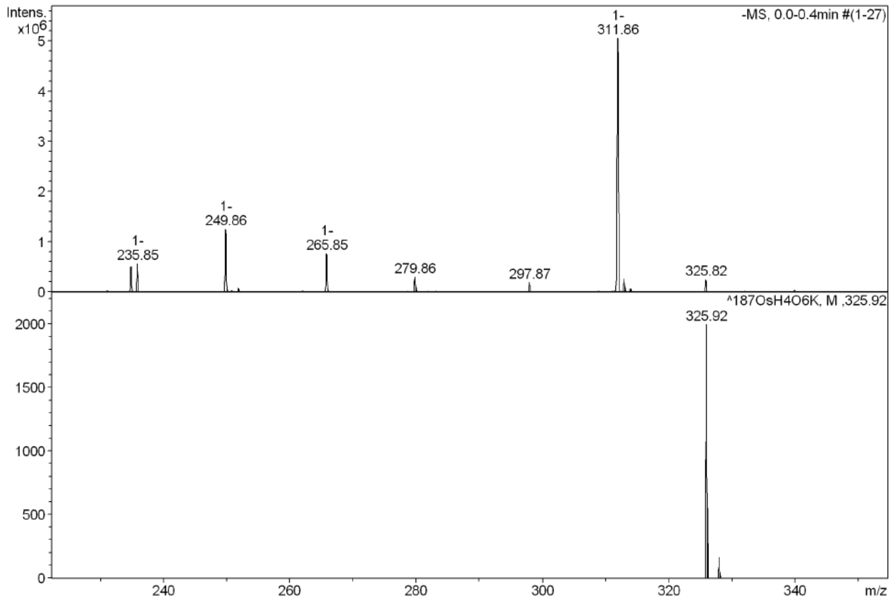

Figure S6. The negative ion ESI mass spectrum of $K_2[^{187}Os^{VI}O_2(OH)_4]$.

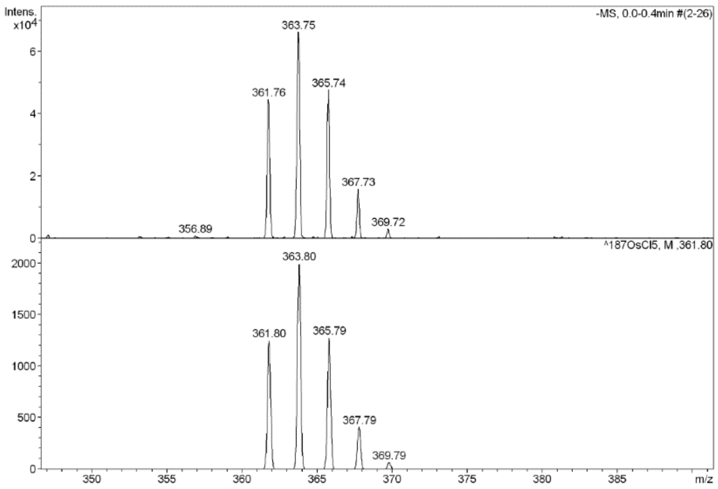

Figure S7. The negative ion ESI mass spectrum of $(H_2pz)_2[^{187}Os^{IV}Cl_6]$.

6. Crystallographic data collection

Table S1. Crystal Data and Details of Data Collection and Refinement for $K_2[^{187}OsO_2(OH)_4]$.

compound	$K_2[^{187}OsO_2(OH)_4]$
empirical formula	$H_4OsO_6K_2$
fw	368.43
space group	*I*4/*mmm*
a, Å	5.5904(2)
b, Å	5.5904(2)
c, Å	9.4276(4)
V [Å^3]	294.64(2)
Z	2
λ [Å]	1.54178
ρ_{calcd}, g cm^{-3}	4.153
cryst size, mm^3	0.10 × 0.07 × 0.07
T [K]	247(2)
μ, mm^{-1}	53.372
$R_1{}^a$	0.0188
$wR_2{}^b$	0.0424
GOF^c	1.003
CCDC no.	2408581

^{a} $R_1 = \Sigma||F_o| - |F_c||/\Sigma|F_o|$.

^{b} $wR_2 = \{\Sigma[w(F_o^2 - F_c^2)^2]/\Sigma[w(F_o^2)^2]\}^{1/2}$.

^{c} GOF = $\{\Sigma[w(F_o^2 - F_c^2)^2]/(n - p)\}^{1/2}$, where n is the number of reflections and p is the total number of parameters refined.
